# Potential Reporting Bias in fMRI Studies of the Brain

**DOI:** 10.1371/journal.pone.0070104

**Published:** 2013-07-25

**Authors:** Sean P. David, Jennifer J. Ware, Isabella M. Chu, Pooja D. Loftus, Paolo Fusar-Poli, Joaquim Radua, Marcus R. Munafò, John P. A. Ioannidis

**Affiliations:** 1 Division of General Medical Disciplines, Department of Medicine, Stanford University School of Medicine, Stanford, California, United States of America; 2 SRI International, Center for Health Science, Policy Division, Menlo Park, California, United States of America; 3 Department of Experimental Psychology, University of Bristol, Bristol, United Kingdom; 4 Institute of Psychological Medicine and Clinical Neurosciences, Cardiff University, Cardiff, United Kingdom; 5 Department of Psychosis Studies, Institute of Psychiatry, King's College London and OASIS team, South London and the Maudsley NHS Foundation Trust, London, United Kingdom; 6 Stanford Prevention Research Center, Department of Medicine, and Department of Health Research and Policy, Stanford University School of Medicine, and Department of Statistics, Stanford University School of Humanities and Sciences, Stanford, California, United States of America; University College London, United Kingdom

## Abstract

**Background:**

Functional magnetic resonance imaging (fMRI) studies have reported multiple activation foci associated with a variety of conditions, stimuli or tasks. However, most of these studies used fewer than 40 participants.

**Methodology:**

After extracting data (number of subjects, condition studied, number of foci identified and threshold) from 94 brain fMRI meta-analyses (*k* = 1,788 unique datasets) published through December of 2011, we analyzed the correlation between individual study sample sizes and number of significant foci reported. We also performed an analysis where we evaluated each meta-analysis to test whether there was a correlation between the sample size of the meta-analysis and the number of foci that it had identified. Correlation coefficients were then combined across all meta-analyses to obtain a summary correlation coefficient with a fixed effects model and we combine correlation coefficients, using a Fisher’s z transformation.

**Principal Findings:**

There was no correlation between sample size and the number of foci reported in single studies (r = 0.0050) but there was a strong correlation between sample size and number of foci in meta-analyses (r = 0.62, p<0.001). Only studies with sample sizes <45 identified larger (>40) numbers of foci and claimed as many discovered foci as studies with sample sizes ≥45, whereas meta-analyses yielded a limited number of foci relative to the yield that would be anticipated from smaller single studies.

**Conclusions:**

These results are consistent with possible reporting biases affecting small fMRI studies and suggest the need to promote standardized large-scale evidence in this field. It may also be that small studies may be analyzed and reported in ways that may generate a larger number of claimed foci or that small fMRI studies with inconclusive, null, or not very promising results may not be published at all.

## Introduction

Since the early 1990s, the use of functional magnetic resonance imaging (fMRI) has become one of the best imaging instruments to assess *in vivo* brain activity. Because the procedure does not require contrast or expose the participants to radiation, the same individual can be scanned repeatedly which alleviates ethical concerns about giving research participants unnecessary scans and safety concerns about repeated scans in clinical populations [1], [2], [3] and resolves many practical issues associated with older functional imaging techniques such as single photon emitted computed tomography or positron emission tomography. This wide acceptance has resulted in a proliferation of fMRI research studies and elucidated regional activation networks associated with a variety of behaviors, neuropsychiatric conditions and brain functions. Consequently, in the last decade there has been an increasing body of meta-analyses looking at these fMRI studies [1], [2] with the aim of reconciling contrasting or inconclusive results across each finding. In particular, the availability of new whole-brain techniques such as Activation Likelihood Estimation (ALE) [4] or Signed Differential Mapping (SDM) [5] has prompted researchers to test the significance of individual findings at the meta-analytical level. Many fMRI research studies find significant associations between activation of a large number of selected foci and the assigned stimuli or condition [6], [7], [8], [9], [10], [11], [12], [13]. While it is reasonable that particular regions of the brain are indeed activated during a given task, it is also possible that positive findings are more likely to be reported, either because investigators are more likely to report them or because journals are more likely to publish positive findings [14], [15], [16]. Our study probes whether there is evidence of such bias in this important and rapidly expanding literature.

A previous empirical evaluation of bias in region of interest (ROI) studies of brain volume abnormalities [16] used an excess significance test based upon observed and expected number of studies with statistically significant results. It found that excess-significance bias was present in this literature, i.e. too many single studies had reported statistically significant results, beyond what would be expected given their small, underpowered sample size, even if brain volume abnormalities did exist. In the present investigation, we sought to examine potential bias in whole-brain fMRI studies. Whole brain imaging studies rely on automated analytical techniques and thus tend to be considered less affected by selective reporting of results or publication biases. However this has never been tested *a priori*. To evaluate bias in whole-brain fMRI studies and the extent of potential over-reporting of significant results we adopted an alternative approach as compared to the previous study based on structural literature. In contrast to the common unit of measurement in structural MRI studies of brain volume, effect sizes for fMRI studies are reported in a variety of ways (e.g., signal change, contrasts of parameter estimates, *Z* scores, and voxel counts, etc.) with limited data repositories of standardized effect sizes for given paradigms. Consequently, our approach was to first examine whether sample size was correlated with the number of foci detected across 1,778 studies that had been included in 94 meta-analyses of whole-brain fMRI. Our first hypothesis was that larger studies would be expected to detect more foci than smaller studies, unless biases tended to inflate the number of foci reported from published smaller studies. Our second hypothesis was that the number of foci would be smaller in single studies than in meta-analyses, unless biases were present, in which case single studies would report more foci than one would expect given their underpowered sample sizes. Finally, we examined whether the use of different thresholds for claiming the discovery of a focus may affect the number of claimed foci, to determine if meta-analyses that used more lenient thresholds identified a larger number of foci.

## Methods

### Search Strategy

**Figure 1:**
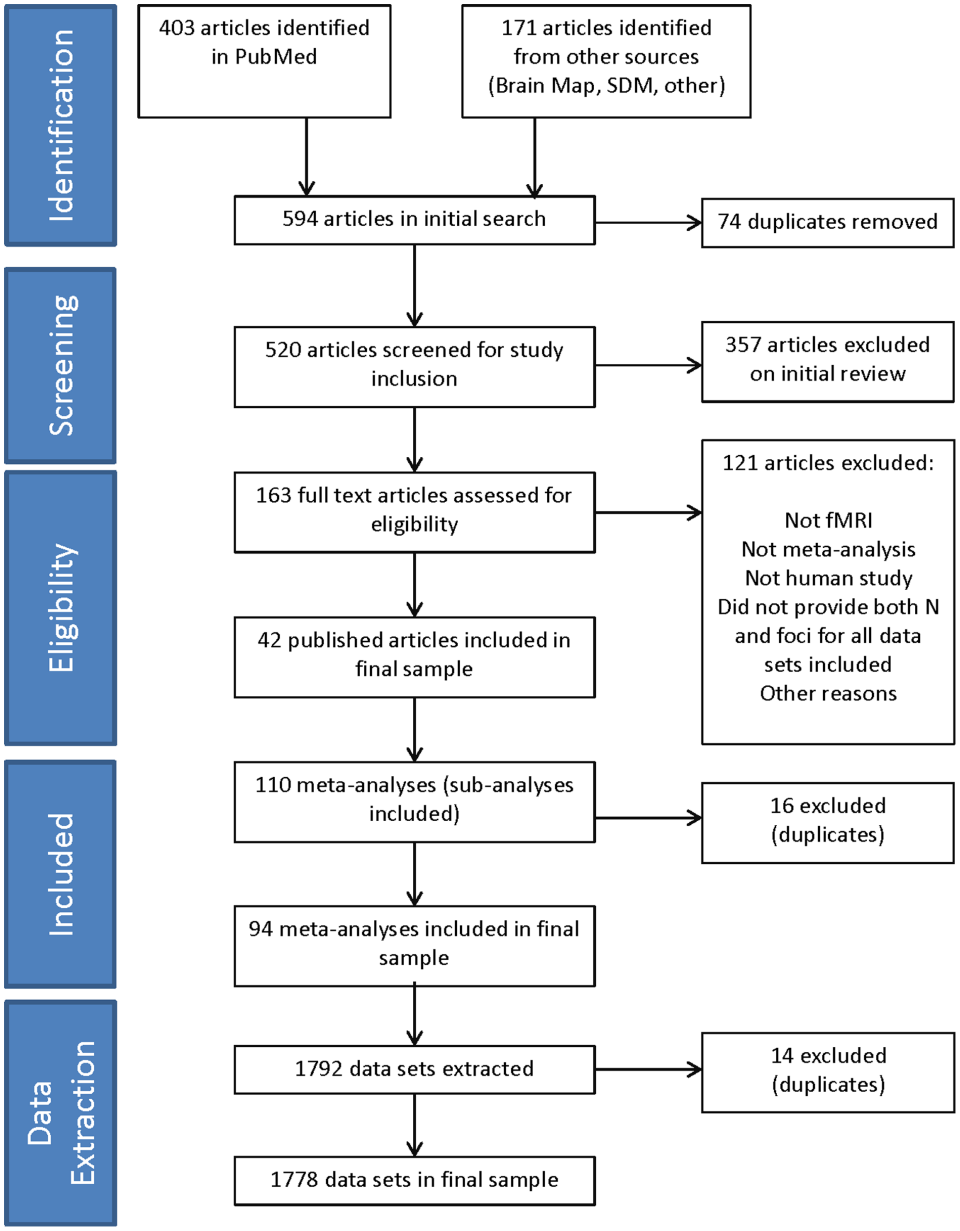
PRISMA Flow chart of literature search and data extraction. Flow chart of literature search, selected meta-analyses papers, selection of sub-analyses and selection of final data sets.

We conducted a two-step literature search. First, we searched on PubMed using the Boolean terms “functional magnetic resonance imaging” and “brain” and “meta-analysis”. All papers listed in PubMed English language papers prior to December 31, 2011 were included. In a second step we also searched the bibliographies of Brain Map (http://brainmap.org/pubs/) and SDM databases (http://sdmproject.com) (last search performed on March 7, 2012). All eligible papers were included regardless of date of publication. After an initial culling of ineligible and duplicate articles, full texts were pulled for all potentially eligible articles. These articles were then hand searched for inclusion criteria and selected by two analysts independently (SPD & PFP), with any discrepancies adjudicated until 100% rater agreement was achieved. To achieve a high standard of reporting we have adopted ‘Preferred Reporting Items for Systematic Reviews and Meta-Analyses’ (PRISMA) guidelines [17] (see Figure 1 below).

Many of the included papers conducted meta-analyses for more than one condition (sub-meta-analyses). These sub-meta-analyses were each considered separately for inclusion in our study. In the case where the same paper included both an overall meta-analysis and separate sub-analyses for different conditions, the sub-analyses were preferentially included (with the overall meta-analysis being considered a duplicate and excluded). To further avoid inclusion of overlapping data, when two meta-analyses included the same subjects observed doing the same or very similar condition (e.g., included the same published study), we retained only the more recent meta-analysis. The unique DOI or PMID was then pulled for each individual study included in these meta-analyses and compiled and compared a second time to eliminate overlapping data sets. If an individual study was included in two meta-analyses where the task performed was judged to be the same (or very similar), this individual study was excluded from the older of the two meta-analyses to insure that no individual study was double counted for the same subjects and task.

### Inclusion Criteria

Meta-analyses were included in our empirical evaluation if: (i) they were whole brain meta-analyses of blood-oxygen-level-dependent (BOLD) fMRI response of human brain; (ii) had listed both the number of subjects and number of foci identified in each individual fMRI study included. Meta-analyses were eligible regardless of the stimuli (i.e. active task or resting state) or neuropsychiatric condition investigated.

Meta-analyses including positron emission topography or other functional brain imaging modalities were included only if they included fMRI studies as well. Exclusion criteria were meta-analyses of Regions of Interest (ROI, not whole brain) - unless these were meta-analyses of whole-brain voxel-based data focusing on large regions (e.g., cerebellum), other fMRI modalities (e.g., diffusion tensor imaging, functional connectivity techniques), or non-human or non-brain studies. If a meta-analysis did not report the number of subjects or foci identified in each individual study it was not included.

### Data Extraction

The total number of study subjects, the condition associated with each of the foci identified, the number of foci identified both for individual studies and the number of foci identified at the meta-analysis level were extracted. We also recorded the threshold used (false discovery rate, *p*-value, or other) for highlighting foci in each meta-analysis.

### Statistical Analyses

We used a Spearman correlation coefficient to evaluate the association between individual study sample sizes and number of significant foci reported in the study. We expected to see a positive correlation, as studies with larger sample size would mean greater power to detect relevant foci. However, this correlation may be close to zero if small studies discover a spuriously large number of foci. This analysis may also be affected by the fact that the individual studies belong to many different meta-analyses focusing on a wide variety of conditions. Therefore we also performed an analysis where we evaluated each meta-analysis to test whether there was a correlation between the sample size of the meta-analysis and the number of foci that it had identified. These correlation coefficients were then combined across all meta-analyses to obtain a summary correlation coefficient with a fixed effects model [13]. To combine correlation coefficients, we used the Fisher’s z transformation [13].

**Figure 2:**
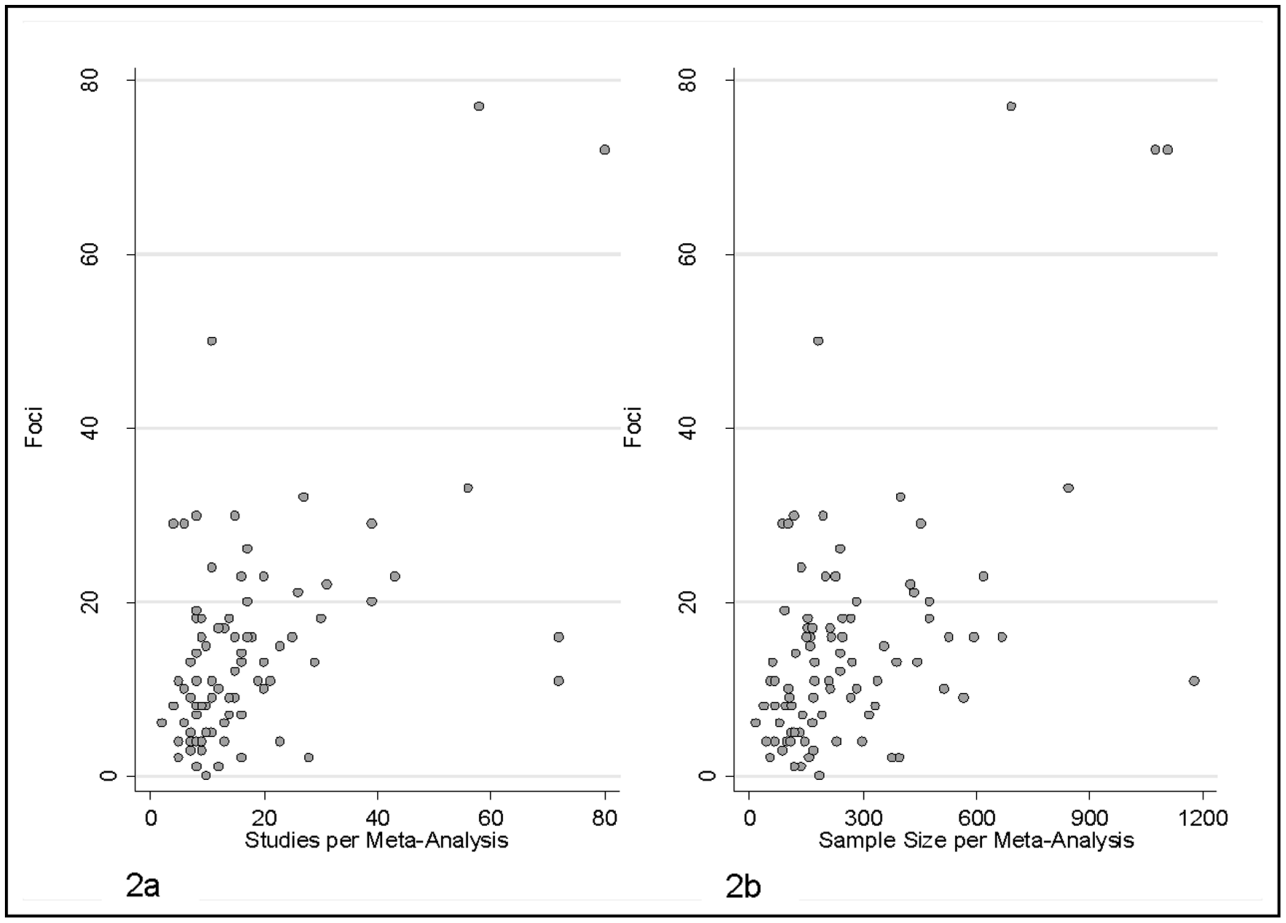
Relationship Between Number of Meta-Analytic Foci, Sample Size and Number of Studies per Meta-Analysis. 2A - Scatter plot of foci (per meta-analysis) and studies per meta-analysis. 2B - scatter plot of foci (per meta-analysis) and sample size per meta-analysis.

We also used Spearman correlation analyses to test whether more foci were identified in a meta-analysis when that meta-analysis had accumulated a larger amount of evidence. We used the number of individual studies and the total number of subjects reported in each of these as measures of the amount of evidence (Figure 2).

Next, we examined whether the use of different thresholds for claiming the discovery of a focus may affect the number of claimed foci to determine if meta-analyses that used more lenient thresholds identified a larger number of foci. Meta-analyses generally did not report the thresholds for individual studies. Given that thresholds for individual studies were rarely reported in meta-analyses, we sampled 2% of the datasets and examined the thresholds used in these studies to understand and describe the caveats about thresholds which may lead to flexibility in the number of foci identified and may be a contributing factor to a large number of foci identified in small individual studies. For meta-analyses, the threshold used in the analysis was almost always reported, and in the large majority this was a *p*-value rather than false discovery rate (FDR) *q*-value. Thus we could assess whether the number of foci identified in a meta-analysis was larger when the most lenient threshold had been used (*p*<0.05) versus when a more stringent threshold (*p*<0.001 or *p*<0.0001) had been used. The number of foci identified in the two groups of meta-analyses was compared using a t-test and was also evaluated as the dependent variable in a linear regression with group (0 if *p*<0.05, 1 if *p*<0.001 or *p*<0.0001) and total sample size of the meta-analysis as the independent variables. All analyses were conducted using Stata^MP^ version 12.

### Sensitivity Analysis

To investigate the possibility that our finding was driven by the convention of reporting only the first three foci in a large cluster we selected 30 papers with fewer than 45 participants and 10 papers with 45 or more participants at random and noted the statistical software used, the number of clusters identified and how many foci per cluster were reported.

### Simulations

It must be noted that some particularities in the way authors report voxel-based fMRI results could conceal the expected positive relationship between the sample size and the number of reported foci. Statistically significant voxels are usually grouped in clusters of spatially contiguous voxels, and only the local maxima (i.e. foci) are reported. Importantly, an increase of the sample size helps non-significant voxels between two close clusters to achieve statistical significance, thus sometimes converting the two close clusters into a single larger one. The number of foci should not be affected by this conversion, but some authors use to report only three foci per cluster. In other words, these authors would report six foci when describing the two close clusters, whilst only three when describing the single larger cluster obtained after an increase of the sample size. In that case, the relationship between the sample size and the number of foci could be downwards biased. A simulation framework was therefore used to assess whether such potential bias could significantly affect the expected relationship. First, 42000 individually-fitted BOLD datasets were simulated by adding normally distributed noise to the gray matter of a brain template of the regions known to activate during a working memory task [18]. Second, these data were smoothed with a relative large Gaussian kernel (σ = 4mm, FWHM = 9-10mm), thus simulating both the spatial covariance observable in raw data and the narrower smoothing usually applied in fMRI pre-processing. Finally, individuals were grouped in 400 simulated studies with different numbers of participants (from *n* = 10 to 200), and standard group-level voxel-based statistics were performed (uncorrected *p* = 0.001, 10 voxels extent).

## Results

### Eligible Meta-analyses and Datasets

The initial search in PubMed returned 403 papers. The search into the SDM and ALE databases contained 167 and 24 papers respectively for a total of 594 articles. After eliminating ineligible articles by abstract, full texts were then pulled for 163 articles and after hand searching for inclusion criteria a final sample was extracted from 42 eligible published papers.

After removal of duplicate meta-analyses, the final sample consisted of 94 separate meta-analyses, which included 1,778 unique data sets (individual whole brain fMRI studies). Appendix Table S1 summarizes data from the 94 meta-analyses. The search strategy for meta-analyses and datasets is summarized in Figure 1. Overall, the median (IQR) was 12.5 (8 to 20) for the number of individual studies per meta-analysis, 13 (10 to 17) for the sample size per individual study, 191 (123 to 355) for the total sample size per meta-analysis, 8 (4 to 15) for the number of foci identified per individual study, and 12.5 (7 to 18) for the number of foci identified per meta-analysis.

### Correlation of amount of Evidence with Number of Identified Foci in Single Studies

**Figure 3:**
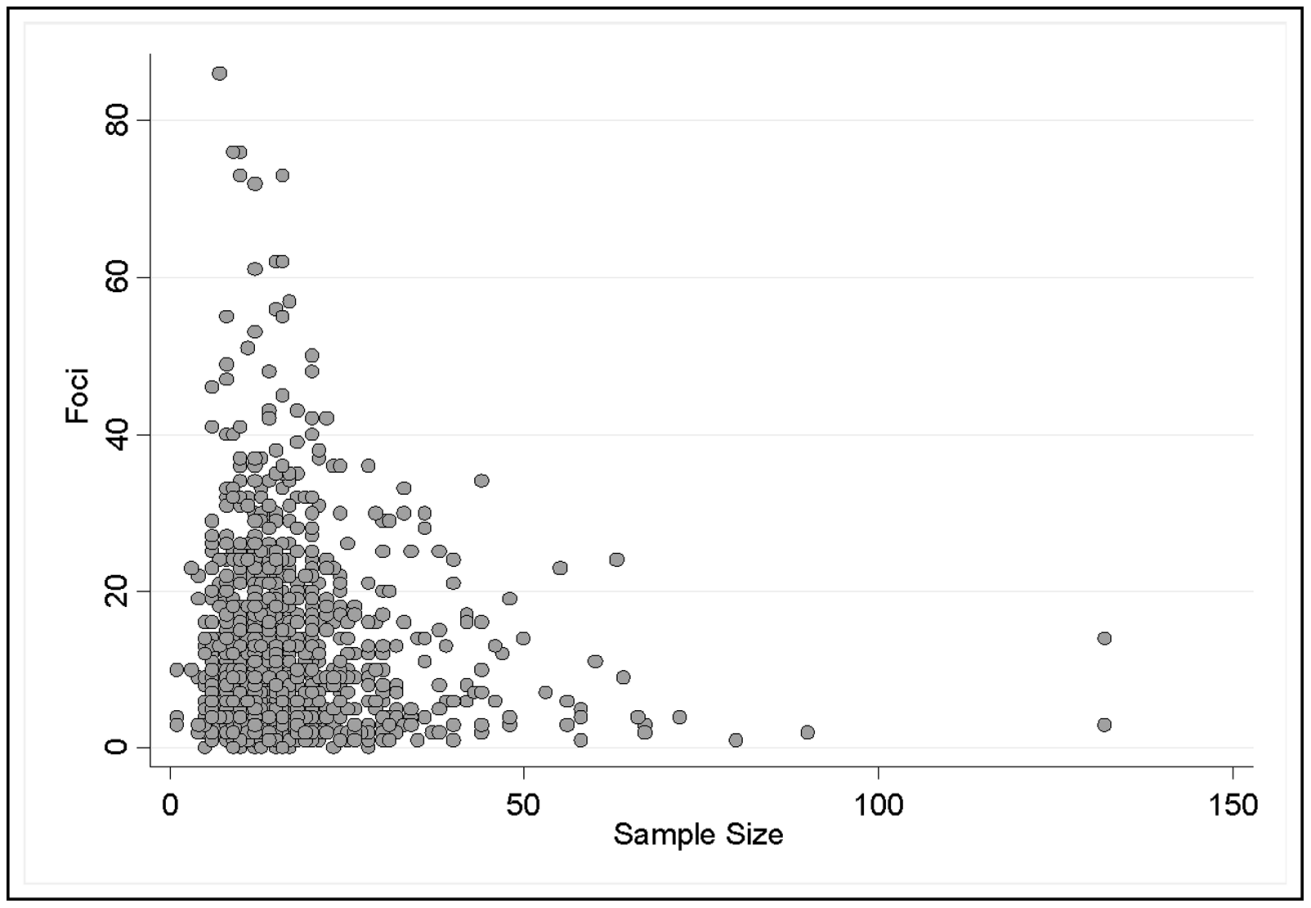
Relationship Between Number of Foci and Sample Size per Study. Scatter plot of foci and sample size from all data sets (N = 1778) within all meta-analyses.

There was no correlation between the sample size of individual studies and the number of foci identified. This was true both when all 1,778 individual studies/datasets were considered independently (*r = *0.0027, 95% CI, −0.045 to 0.050, *p* = 0.86, Figure 3) as well as when correlations were first calculated within each meta-analysis and then combined across all 94 meta-analyses (*r* = 0.0050, 95% CI, −0.165 to 0.175, *p* = 0.95). Only six of the 94 meta-analyses showed a within-meta-analysis correlation between individual study sample size and number of foci that was greater than 0.500. This implies that sample size is not a significant determinant of foci detected, and that small individual studies report, on average, a similar number of foci as larger studies. This finding is counterintuitive to power considerations as one would expect the power to detect an increasing number of foci with an increasing number of study participants. Also from Figure 3, it is worth noting that the individual studies that detected the highest number of foci (>40) are typically studies with very small sample sizes (*n*<25), while the individual studies with the largest sample sizes (*n*>45) always identified fewer than 25 foci and most identified fewer than 10. Such irregularities suggest that smaller individual fMRI studies may be subject to selective analysis and reporting biases.

### Correlation of amount of Evidence with Number of Identified Foci in Meta-analyses

As shown in Figure 2, there was a strong correlation between the number of foci identified in a meta-analysis and the total number of individual studies per meta-analysis (*r* = 0.67, *p*<0.001, Figure 2a) as well as cumulative sample size of the meta-analysis (*r* = 0.62, *p*<0.001 Figure 2b). In contrast to the situation with individual studies, the more information included in a meta-analysis, the greater the number of foci it detected.

When comparing Figure 2b (meta-analysis sample size) with Figure 3 (individual study sample size), in the case of meta-analyses, the largest numbers of identified foci correspond to the largest sample sizes, while the pattern was neutral and even reversed in individual studies where the largest number of foci were detected in smaller studies. In the single study plot (Figure 3), 16.77% of the individual fMRI studies with sample size <40 (median n = 12) claimed to identify more than 20 foci. However, in the meta-analysis level plot (Figure 2b), a similar proportion (20%) detected more than 20 foci even though the median sample size was 16-times larger (n = 191). When we compared all meta-analyses to all individual studies, the former had significantly larger sample sizes (mean *n* = 288.3 vs 15.2, *p*<0.001), and identified slightly more foci (mean 15.60 vs 10.71, *p*≤0.001). However, the ratio of foci divided by sample size was markedly smaller (mean.0521 versus 0.8732, *p*<0.001).

Given that statistical packages and analytic techniques for whole-brain statistical parametric mapping (SPM) may have changed over time, we examined the effect of publication year on the relationship between number of reported foci and sample size. However, there was no correlation between the number of foci per sample size and publication year for single studies (*r* = −0.0198, *p* = 0.767) (*n*<45: *r* = 0.0165, *p* = 0.2890; *n*≥45: *r* = 0.02100, *p* = 0.9881) or meta-analyses (*r* = 0. 0250, *p* = 0. 346).

### Selection of Thresholds

As stated in the methods, meta-analyses do not usually report the thresholds used for inclusion of individual studies. Across the 94 meta-analyses, only two reported individual thresholds for included studies. Three of the 94 meta-analyses reported a minimum acceptable threshold for study inclusion, but in two of these cases, a range of acceptable values was given rather than the exact threshold. In the remainder of cases (*k* = 89), no clear cut-off for study inclusion was given.

In 2% of studies sampled (*k = *36 individual fMRI studies) the thresholds were generally *p*<0.001 for individual foci (28 of 36 studies). Three of the individual studies did not give a specific threshold cut-off. Almost all individual studies gave a study-wide correction value of *p*<0.05 (*k* = 22) or *p*<0.01 (k = 5). In the remaining 9 studies a study-wide error threshold was not given.

All the meta-analyses gave a study-wide correction threshold. Most studies (85 of 94) gave this as a *p-*value, most frequently <0.05 (47 of 94 meta-analyses) but also <0.01 (16 meta-analyses), ≤0.001 (17 meta-analyses) or <0.0001 (2 meta-analyses). The remaining 9 meta-analyses expressed the threshold as an FDR (*q* value). The *p*-values (or FDR values) for the 94 meta-analyses are given in Appendix Table S1.

The median ratio of foci claimed divided by sample size for the 47 meta-analyses that used a study-wide corrected *p*-value <0.05 threshold was 0.06 (IQR, 0.04 to 0.08). Conversely, the median ratio of foci divided by sample size for the 22 sampled individual studies that used a study-wide correction value of *p*<0.05 was 0.40 (IQR, 0.22 to 1.10). Thus, even with the standardization of the threshold, the meta-analyses have a significantly smaller foci to sample size ratio compared to individual studies (*p*<0.001 by the Mann-Whitney U test).

In multivariable linear regression, the number of foci claimed by a meta-analysis increased with increasing sample size (coefficient +0.323 [*p*<0.001], per 10 additional subjects included in the meta-analysis), while there was no significant impact from the use of a more lenient threshold (coefficient +0.419 [*p* = 0.88] with study-wide *p*<0.05 than with study-wide *p*<0.001 or <0.0001).

### Sensitivity Analysis

Twenty-two (73%) of the smaller studies (n<45) and six of the larger (n≥45) (60%) papers used SPM. The remaining studies used a variety of other software packages. In 2 cases (1 in each strata) the software was not given. There was considerable heterogeneity in the way clusters and foci (Talairach coordinates) but we did not find evidence of widespread reporting of only the top three local maxima per clusters according to sample size. In the 12 smaller study papers where it was clear how many foci per cluster had been reported, 9 reported only 1 focus per cluster. The remaining three studies reported 2 to 6 foci/cluster with the number of foci largely dependent on the size of the cluster. Among the larger studies, 5 reported 1 focus per cluster, in the remaining five papers it could not be determined how many foci/cluster were reported. These analyses did not indicate that there are fewer clusters in larger studies with more statistical power and that the converse was the case for smaller studies. We did not find evidence of differential reporting methods for small and large studies. When more than one foci per cluster was reported, the number of foci per cluster was largely driven by cluster size.

### Simulation Analysis

**Figure 4:**
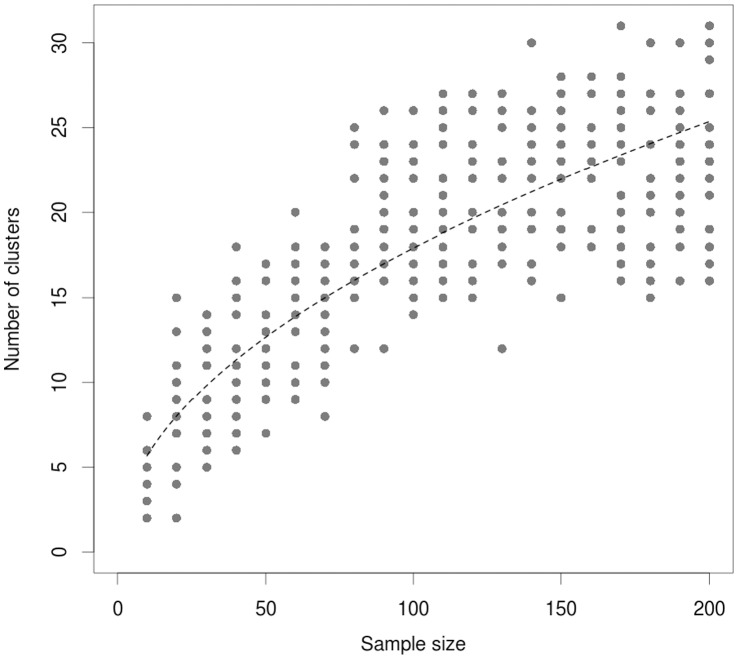
Relationship Between Sample Size and Number of Clusters in Simulated fMRI Data. Relationship between sample size and number of clusters in simulated fMRI data.

As shown in Figure 4, the number of clusters followed a clear positive relationship with the sample size. The relationship would be the same if each cluster were substituted by 3 reported foci. The results demonstrated that the number of clusters were 4, 4, 6, 7, 10, 18, 16, 25, 21 and 24, corresponding simulations for 10, 20, 30, 40, 50, 60, 70, 80, 90 and 100 participants, respectively.

## Discussion

Despite the large body of fMRI literature, most published studies have samples sizes that would be considered small by conventional standards. Nevertheless, the number of foci claimed to be discovered by small studies is relatively large and we found absolutely no correlation between the sample size of a study and the number of foci that it claims. This is counterintuitive to power considerations and it suggests that biases that inflate the number of claimed foci may affect disproportionately the smaller studies in the literature. Consistent with this picture, meta-analyses identified only slightly more total foci than single studies, despite having sample sizes that were almost 20 times larger; thus studies with n<45 identify far more foci per subject than meta-analyses do. This picture persisted when we compared only single studies and meta-analyses that used the same study-wide corrected *p*-value threshold.

This evidence is consistent with the presence of selective reporting biases causing an excess of significance in the published literature. We cannot exclude the possibility that larger studies and even large meta-analyses are also affected by such biases. Different mechanisms may contribute to this excess significance.

First, smaller fMRI studies may be underpowered and inflate the number of reported foci. The average sample size of the retrieved studies was 13 subjects and the vast majority (94%) of individual studies included in the meta-analyses we examined had fewer than 30 patients. FMRI is a powerful technique to investigate subtle neurophysiological brain changes and the adequate sample size depends on the nature of statistical inference requested [19]. An average sample size of 13 subjects is probably well below the optimal sample size for an fMRI study, especially when variability across measurements and patients are considered. Some authors have proposed optimal sample sizes of 16-32 subjects per group [19], [20], [21], suggesting that between-subject comparison studies of *n*<30 are too small even by liberal estimates. A recent commentary by Friston posed “Ten Ironic Rules” that claimed several fallacies to the application of classical inference to sample size and power for functional neuroimaging studies whereby studies with smaller sample sizes generate more reliable data because they are less likely to report findings with trivial effect sizes. Friston’s assertions have been challenged on the basis that weak statistical power comes with lower positive predictive value, increases the likelihood of false positive results [22], [23]. Moreover, a recent paper by authors of the present paper (MM & JPAI) reported power analyses of single studies included in 49 meta-analyses and found that almost 50% of studies had an average power lower than 20% [24].

On the other hand, the presence of many underpowered studies in the available literature may be due to the technical and logistic complexity of fMRI and the cost involved. In theory the field would thus benefit from the conduct of collaborative studies where many centers join forces and generate large sample sizes with standardized data collection, analysis and reporting plans [13]. However, the use of different scanners in multicenter fMRI studies may introduce additional significant heterogeneity [25], [26].

Second, small fMRI studies with inconclusive, null, or not very promising results (e.g. very few foci identified) may not be published at all. For example, recent voxel-based meta-analyses of structural studies in early psychosis [27], [28] uncovered only one study reporting no significant between-group results [29]. Peer-reviews should be as strict when assessing the methods of a study reporting abnormalities in expected brain regions, as when assessing the methods of a study not finding any expectable finding. Similarly, acceptance or rejection of a manuscript should not depend on whether abnormalities are detected or not, or on the specific brain regions found to be abnormal. Publication bias is very difficult to detect in meta-analyses done after the fact, especially when all the published studies are small [30], [31]. Bias is sometimes further extenuated by the fact that some available voxel-based packages can only analyze sets of *x*, *y*, *z* spatial coordinates excluding studies reporting null results.

Third, small studies may be analyzed and reported in ways that may generate a larger number of claimed foci. The analysis option that would cause such an increased number of foci is to use more lenient statistical thresholds for claiming a discovery in smaller studies. In fact, the meta-analytical approach adopted by most packages does not correct the number of foci entered for their statistical significance nor for the sample size of the single study. In other words, foci reported in small studies with liberal statistical threshold are directly compared with foci reported in large studies adopting more stringent thresholds. Only in the most recent years this problem has been recognized and partially overcome in the current version of the two most widely used voxel-based packages (i.e. ALE and SDM) [5], [32]. Additionally, it is not uncommon in neuroimaging studies that the statistical threshold for some regions of interest is rather more liberal than for the rest of the brain. The use of inconsistent and erratic statistical threshold in the same study can affect the number of foci detected. Although we were unable to test this at the level of the individual study, this problem is well recognized in the imaging literature. A recent voxel-based packages such as SDM require the user to carefully check that the same statistical threshold is used throughout the whole brain to avoid biases toward liberally thresholded brain regions [33]. At the level of the meta-analyses, our empirical analysis showed that there is heterogeneity in the selection of statistical thresholds, but this heterogeneity is unlikely to explain the whole bias. In fact, we did not find that meta-analyses with more stringent thresholds (*p*<0.01 or *p*<0.001) reported significantly fewer foci than those with more lenient thresholds (*p*<0.05). The results of our simulation suggest that for a given set of analysis choices for a given task (degree of spatial smoothing, statistical thresholds, etc), increased sample size should increase the number of significant clusters – each containing one or more local maxima (foci). If, however, arbitrary analysis choices account for the larger number of reported foci in smaller studies, it would be consistent with the statement that our conclusion that biases that inflate the number of claimed foci may affect disproportionately the smaller studies in the literature.

Fourth, analyses can be built post-hoc on the basis of the researchers’ hypotheses or on the basis of the most significant result. Although whole-brain analyses are considered less affected by this potential bias than region of interest (ROI) analyses, this has not been explicitly tested. The use of Small Volume Correction (SVC) techniques in addition to standard whole-brain analyses may be used to alter the statistical threshold in selected ROIs, thus impacting on the number of foci reported. For example, insignificant results at the standard whole-brain levels may be published as significant results after SVC. Most of the meta-analyses included in this study did not clarify whether the individual studies performed whole brain analyses or whether they also included SCV coordinates. Recent recommendations in the field (http://sdmproject.com/) [34] clearly indicate that prior to conducting the voxel-based meta-analysis, there should be strict selection of the reported peaks by only including those that appear statistically significant at the whole-brain level (no SVCs). To overcome these problems, authors may also be encouraged to blind the statistical analyses of the imaging datasets to avoid analyses be built post-hoc on the basis of the results. Similarly, all individual studies should explicitly acknowledge the number of analyses performed giving a clear rationale for each, to control for conducting exploratory analyses and reporting the most significant result.

Some limitations should be acknowledged. First, we used the data presented in already available meta-analyses and some of these may have data or analysis errors. However, it would have been prohibitively resource intensive to repeat from scratch 94 meta-analyses and to extract the requisite data in detail from almost two thousand papers. The availability of large-scale evidence from such a large number of meta-analyses offers an excellent launching point for assessing the big picture in this important research field. Second, it is possible that some large studies may be of poor quality and thus to find fewer foci than smaller studies simply because of poor study design and poor measurements. However, this is unlikely to be a systematic problem with large studies, and if anything one would expect higher quality criteria in larger investigations that are typically performed by more experienced teams. Third, the number of foci identified in meta-analyses may also be biased. For example, they can report only activation and not deactivation clusters and it is not an absolute gold standard, since meta-analyses carry with them many of the biases of the studies that they combine. However, it is generally agreed that the findings form meta-analyses have a higher level in the hierarchy of evidence than findings from isolated individual studies. Fourth, there are a number of other factors reflecting a range of potential arbitrary choices in analyses that could affect the number of activation foci generated (e.g., inter-study variation in the degree of spatial smoothing, use of fixed-effects instead of mixed-effects analyses, use of cluster extent-based correction, etc.), but there is no reason to expect that variation in these and other analytic methods would vary by sample size. Fifth, as many studies incorporate repeated-measures designs and/or multiple between-subject groups and others do not, the numbers included in our analyses represent some heterogeneity in study designs with different implications for statistical power. However, the sample sizes included in our analyses represented the number of subjects per group for first-level analyses (within-subject task/stimulus contrasts) that were incorporated into meta-analyses. While we can not definitively exclude the possibility that some repeated-measures analyses were included in the foci counts, we have no reason to expect that larger sample sizes would not improve positive predictive value for group comparisons or higher-level, repeated-measures analyses.

Finally, while it is possible that our finding is partially driven by the likelihood that larger studies identify fewer clusters due to smoothing, and thus, are likely to report fewer foci, this would still support our finding that that smaller studies are likely to report more foci. Whether this is the result of smoothing or because of a number of analysis choices that may be influenced by sample size is difficult to determine.

### Conclusions

Acknowledging these caveats, the emerging picture is consistent with the presence of excess significance biases in the literature of whole brain fMRI affecting predominantly smaller studies. Similar biases have been identified in a large number of other fields with different methods [16], [35], [36]. Improvements in standardization of research in this field with delineation of acceptable and optimal practices may be useful. Efforts at generating large-scale systematic evidence may be instrumental in improving the yield of information in this important research field.

## Supporting Information

Table S1 **Meta-analysis papers included in correlational analyses**
(DOCX)
