# Supplementary material for: Potential Reporting Bias in fMRI Studies of the Brain
Source: PLoS One. 2013 Jul 25;8(7):e70104. doi: 10.1371/journal.pone.0070104 (PMC3723634; doi:10.1371/journal.pone.0070104)
Supplement: Table S1 — Meta-analysis papers included in correlational analyses (DOCX) [file pone.0070104.s001.docx]

**Supplemental Table 1: Meta-analysis papers included in correlational analyses**

| **First author (Year) & Citation** | **Topic, disease and/or condition (stimulus, task or resting state)** | **Number of studies** | **Total sample size (range per MA)** | **MA foci (range of foci per study)** | **Meta-analysis-wide threshold**  **(*p*-value or FDR)** |
| --- | --- | --- | --- | --- | --- |
| Albrecht (2010)[^1^](#_ENREF_1) | Trigeminal stimulation | 9 | 87 (8 to 30) | 29 (10 to 42) | *p*<0.05 |
| Albrecht (2010)[^1^](#_ENREF_1) | Trigeminal vs. olfactory stimulation | 8 | 117 (8 to 22) | 30 (5 to 32) | *p*<0.05 |
| Amanzio (2011)[^2^](#_ENREF_2) | Placebo analgesia | 35 | 402 (9 to 31) | 32 (0 to 14) | *p*<0.05 |
| Arsalidou (2011)[^3^](#_ENREF_3) | Facial expressions | 11 | 140 (8 to 20) | 24 (5 to 27) | *p*<0.01 |
| Brooks (2012)[^4^](#_ENREF_4) | Physiological arousal | 7 | 107 (11 to 20) | 9 (5 to 62) | *p*<0.05 |
| Brooks (2012)[^4^](#_ENREF_4) | Facial arousal | 12 | 217 (8 to 56) | 10 (6 to 55) | *p*<0.05 |
| Brooks (2012)[^4^](#_ENREF_4) | Word arousal | 10 | 187 (3 to 36) | 0 (4 to 30) | *p*<0.05 |
| Brooks (2011)[^4^](#_ENREF_4) | Miscellaneous audio arousal | 3 | 126(9 to 20) | 14 (2 to 28) | *p*<0.05 |
| Brown (2011)[^5^](#_ENREF_5) | Aesthetic auditory appraisal | 8 | 98 (10 to 19) | 8 (2 to 37) | *p*<0.05 |
| Brown (2012)[^5^](#_ENREF_5) | Aesthetic gustatory appraisal | 16 | 241 (6 to 67) | 14 (2 to 19) | *p*<0.05 |
| Brown (2011)[^5^](#_ENREF_5) | Aesthetic olfactory appraisal | 13 | 167 (6 to 28) | 6 (2 to 36) | *p*<0.05 |
| Brown (2011)[^5^](#_ENREF_5) | Aesthetic visual appraisal | 56 | 845 (6 to 48) | 33 (1 to 86) | *p*<0.05 |
| Bzdok (2011)[^6^](#_ENREF_6) | Facial expressions | 16 | 390 (13 to 48) | 13 (1 to 21) | *p*<0.05 |
| Caspers (2010)[^7^](#_ENREF_7) | Hand motor activity | 104 | 2067 (6 to 58) | n/a (2 to 72) | *p*<0.05 |
| Davis (2009)[^8^](#_ENREF_8) | Pseudowords vs words | 11 | 184 (11 to 28) | 50 (0 to 7) | *p*<0.0038 |
| Davis (2009)[^8^](#_ENREF_8) | Words vs pseudowords | 11 | 184 (11 to 28) | 50 (0 to 33) | *p*<0.0038 |
| Derrfuss (2005)[^9^](#_ENREF_9) | Task-switching | 16 | 172 (6 to 16) | 13 (1 to 13) | *p*<0.0001 |
| Di Martino (2009)[^10^](#_ENREF_10) | Social processes (multiple stimulus/task conditions) (autism spectrum disorder [ASD] cases vs controls) | 24 | 453 (11 to 14) | 29 (2 to 82) | *p*<0.05 |
| Di Martino (2009)[^10^](#_ENREF_10) | Non-social processes (multiple stimulus/task conditions) (ASD cases vs controls) | 15 | 479 (12 to 36) | 20 (9 to 41) | *p*<0.05 |
| Diekhof (2011)[^11^](#_ENREF_11) | Negative affect regulation during fear extinction | 10 | 170 (10 to 35) | 3 (1 to 16) | *p*<0.05 |
| Diekhof (2011)[^11^](#_ENREF_11) | Negative affect regulation during placebo control | 14 | 271 (9 to 43) | 9 (2 to 24) | *p*<0.05 |
| Diekhof (2011)[^11^](#_ENREF_11) | Negative affect regulation during fear reappraisal | 25 | 529 (10 to 56) | 16 (1 to 37) | *p*<0.05 |
| Eickhoff (2009)[^12^](#_ENREF_12) | Hand movements | 38 | 672 (5 to 15) | 16 (1 to 27) | *p* <0.05 |
| Gianaros (2009)[^13^](#_ENREF_13) | Blood pressure reactivity | 4 | 104 (6 to 46) | 29 (6 to 23) | *p*<0.0038 |
| Houdè (2010)[^14^](#_ENREF_14) | Numerical processing | 7 | 88 (8 to 19) | 3 (3 to 19) | *p*<0.001 |
| Houdè (2010)[^14^](#_ENREF_14) | Executive function (children only) | 13 | 318 (6 to 30) | 7 (4 to 35) | *p*<0.001 |
| Houdè (2010)[^14^](#_ENREF_14) | Executive function (adolescents only) | 15 | 195 (9 to 29) | 7 (3 to 31) | *p*<0.001 |
| Houdè (2010)[^14^](#_ENREF_14) | Reading | 15 | 241 (5 to 64) | 12 (1 to 20) | *p*<0.001 |
| Jardri (2011)[^15^](#_ENREF_15) | Auditory verbal hallucinations (schizophrenia spectrum disorder cases only) | 10 | 69 (1 to 24) | 8 (3 to 23) | *p*<0.05 |
| Jirak (2010)[^16^](#_ENREF_16) | Sensorimotor language processing | 21 | 342 (12 to 22) | 11 (5 to 62) | *p*<0.01 |
| Kim (2010)[^17^](#_ENREF_17) | Working memory (pictorial recall vs knowing) | 12 | 217 (11 to 44) | 17 (4 to 30) | *p*<0.05 |
| Kim (2010)[^17^](#_ENREF_17) | Working memory (pictorial/lexical recall vs forgetting) | 72 | 1177 (9 to 30) | 11 (1 to 42) | *p*<0.05 |
| Kim (2010)[^17^](#_ENREF_17) | Working memory (pictorial & lexical forgetting vs recall) | 17 | 286 (11 to 30) | 20 (2 to 15) | *p*<0.05 |
| Krain (2006)[^18^](#_ENREF_18) | Risky decision making | 15 | 218 (8 to 20) | 16 (1 to 28) | *p*<0.05 |
| Krain (2006)[^18^](#_ENREF_18) | Ambiguous decision making | 14 | 248 (5 to 30) | 18 (2 to 29) | *p*<0.05 |
| Kuhn (2011)[^19^](#_ENREF_19) | Sexual cue reactivity (heterosexual males only) | 8 | 154 (10 to 44) | 18 (13 to 43) | *p*<0.01 |
| Kuhn (2011)[^19^](#_ENREF_19) | Sexual cue reactivity cross-correlation with penile turgidity (heterosexual males only) | 8 | 39 (10 to 44) | 8 (13 to 43) | *p*<0.01 |
| Kuhn (2011)[^20^](#_ENREF_20) | Resting state brain activity (schizophrenia vs healthy controls) | 11 | 567 (25 to 132) | 9 (1 to 28) | *p*<0.01 |
| Kuhn (2011)[^20^](#_ENREF_20) | Resting state brain activity (depression vs healthy controls) | 12 | 515 (12 to 90) | 10 (2 to 13) | *p*<0.01 |
| Kuhn (2011)[^21^](#_ENREF_21) | Smoking cue reactivity | 13 | 231 (8 to 42) | 4 (3 to 22) | *p*<0.01 |
| Kuhn (2011)[^21^](#_ENREF_21) | Alcohol cue reactivity | 12 | 112 (4 to 24) | 8 (2 to 21) | *p*<0.01 |
| Kuhn (2011)[^21^](#_ENREF_21) | Cocaine cue reactivity | 4 | 83 (8 to 24) | 6 (1 to 9) | *p*<0.01 |
| Laird (2010)[^22^](#_ENREF_22) | Working memory (encoding recall) | 16 | 240 (6 to 24) | 26 (4 to 61) | *p*<0.05 |
| Laird (2010)[^22^](#_ENREF_22) | Working memory (paired associate recall) | 16 | 205 (6 to 24) | 23 (4 to 61) | *p*<0.05 |
| Maisog (2008)[^23^](#_ENREF_23) | Lexical stimuli (dyslexia patients vs controls) | 9 | 398 (10 to 34) | 2 (4 to 18) | **p*<0.05 |
| Maisog (2008)[^23^](#_ENREF_23) | Lexial stimuli (controls vs dyslexia patients) | 6 | 286 (12 to 34) | 10 (1 to 31) | **p*<0.05 |
| Mar (2011)[^24^](#_ENREF_24) | Theory-of-mind (non-story based) | 20 | 274 (6 to 25) | 13 (4 to 19) | *p*<0.05 |
| Mar (2011)[^24^](#_ENREF_24) | Theory-of-mind (story-based) | 43 | 623 (5 to 33) | 23 (1 to 53) | *p*<0.05 |
| Mar (2011)[^24^](#_ENREF_24) | Story comprehension | 23 | 355 (5 to 30) | 15 (2 to 21) | *p*<0.05 |
| Mechias (2009)[^25^](#_ENREF_25) | Instructed fear conditioning | 10 | 162 (8 to 42) | 15 (2 to 56) | FDR <0.01 |
| Mechias (2009)[^25^](#_ENREF_25) | Uninstructed fear conditioning | 10 | 198 (8 to 42) | 30 (2 to 56) | FDR<0.01 |
| Mohr (2010)[^26^](#_ENREF_26) | Decision risk | 21 | 446 (5 to 25) | 13 (2 to 32) | FDR<0.05 |
| Moulton (2010)[^27^](#_ENREF_27) *** | Painful stimuli | 57 | 695 (4 to 47) | 77 (0 to 32) | *p*<0.001 |
| Petacchi (2005)[^28^](#_ENREF_28) | Auditory functioning (passive & active listening) | 15 | 174 (4 to 18) | 11 (2 to 34) | *p*<0.01 |
| Petacchi (2005)[^28^](#_ENREF_28) | Auditory functioning (passive listening) | 10 | 71 (4 to 18) | 11 (2 to 22) | *p*<0.01 |
| Richlan (2009)[^29^](#_ENREF_29) | Reading (dyslexia patients only) | 17 | 595 (10 to 72) | 16 (2 to 25) | FDR <0.05 |
| Richlan (2011)[^30^](#_ENREF_30) | Reading (dyslexic children only) | 9 | 336 (28 to 66) | 8 (2 to 14) | ***p*<0.001 |
| Richlan (2011)[^30^](#_ENREF_30) | Reading (dyslexic adults only) | 9 | 271 (10 to 72) | 18 (1 to 33) | ***p*<0.001 |
| Rotge (2008)[^31^](#_ENREF_31) | Obsessive-compulsive disease (OCD) symptoms (OCD patients only) | 8 | 94 (4 to 33) | 19 (1 to 30) | *p*<0.01 |
| Simmonds (2008)[^32^](#_ENREF_32) | Working memory (Go/No-Go) (simple) | 5 | 110 (11 to 48) | 4 (3 to 21) | *p*<0.001 |
| Simmonds (2008)[^32^](#_ENREF_32) | Working memory (Go/No-Go) (complex) | 6 | 102 (14 to 28) | 10 (3 to 23) | *p*<0.001 |
| Simmonds (2008)[^32^](#_ENREF_32) | Working memory (Go/No-Go) (simple & complex) | 11 | 212 (11 to 48) | 11 (3 to 23) | *p*<0.001 |
| Sörös (2009)[^33^](#_ENREF_33) | Swallowing (water) | 7 | 65 (8 to 14) | 13 (5 to 32) | FDR<0.05 |
| Sörös (2009)[^33^](#_ENREF_33) | Swallowing (saliva) | 5 | 56 (8 to 14) | 11 (5 to 24) | FDR<0.05 |
| Spaniol (2009)[^34^](#_ENREF_34) | Working memory (episodic encoding) | 26 | 435 (12 to 25) | 21 (1 to 37) | *p*<0.05 |
| Spaniol (2009)[^34^](#_ENREF_34) | Working memory (episodic retrieval) | 30 | 478 (8 to 32) | 18 (1 to 34) | *p*<0.05 |
| Spaniol (2009)[^34^](#_ENREF_34) | Working memory (objective recollection) | 12 | 167 (8 to 21) | 17 (1 to 31) | *p*<0.05 |
| Spaniol (2009)[^34^](#_ENREF_34) | Working memory (subjective recollection) | 9 | 153 (11 to 28) | 16 (2 to 30) | *p*<0.05 |
| Spreng (2009)[^35^](#_ENREF_35) | Resting state compared to multiple active tasks | 20 | 228 (5 to 132) | 23 (1 to 22) | *p*<0.05 |
| Spreng (2009)[^35^](#_ENREF_35) | Cued prospection | 6 | 154 (10 to 21) | 17 (6 to 24) | *p*<0.05 |
| Spreng (2009)[^35^](#_ENREF_35) | Autobiographical memory recall | 20 | 426 (5 to 24) | 22 (4 to 24) | *p*<0.05 |
| Spreng (2009)[^35^](#_ENREF_35) | Navigation tasks in mental environment | 13 | 555 (5 to 20) | 17 (3 to 19) | *p*<0.05 |
| Spreng (2009)[^35^](#_ENREF_35) | Theory of mind | 31 | 84 (6 to 32) | 22 (1 to 18) | *p*<0.05 |
| Spreng (2010)[^36^](#_ENREF_36) | Working memory (encoding stimuli) (younger adults) | 80 | 1078 (5 to 30) | 72 (0 to 73) | *p*<0.05 |
| Spreng (2010)[^36^](#_ENREF_36) | Working memory (older adults) | 80 | 1106 (6 to 40) | 72 (2 to 55) | *p*<0.05 |
| Stoodley (2009)[^37^](#_ENREF_37) *** | Motor function | 7 | 71 (8 to 13) | 4 (1 to 12) | *p*=0.001 |
| Stoodley (2009)[^37^](#_ENREF_37) *** | Somatosensory & spatial processing | 11 | 19 (8 to 24) | 6 (1 to 6) | *p*=0.001 |
| Stoodley (2009)[^37^](#_ENREF_37) *** | Spatial processing | 11 | 130 (6 to 22) | 5 (1 to 14) | *p*=0.001 |
| Stoodley (2009)[^37^](#_ENREF_37) *** | Language processing | 11 | 137 (6 to 22) | 5 (1 to 14) | *p*=0.001 |
| Stoodley (2009)[^37^](#_ENREF_37) *** | Working memory | 8 | 144 (11 to 30) | 7 (1 to 8) | *p*=0.001 |
| Stoodley (2009)[^37^](#_ENREF_37) *** | Executive functioning | 8 | 99 (6 to 20) | 4 (2 to 10) | *p*= 0.001 |
| Stoodley (2009)[^37^](#_ENREF_37) *** | Emotional processing | 9 | 149 (6 to 38) | 4 (1 to 5) | *p*= 0.001 |
| Takai (2010)[^38^](#_ENREF_38) | Respiration | 7 | 44 (5 to 10) | 4 (2 to 26) | *p*<0.01 |
| Takai (2010)[^38^](#_ENREF_38) | Lip movement | 10 | 123 (6 to 30) | 5 (2 to 30) | *p*<0.01 |
| Takai (2010)[^38^](#_ENREF_38) | Swallowing | 25 | 158 (7 to 14) | 2 (5 to 34) | *p*<0.01 |
| Takai (2010)[^38^](#_ENREF_38) | Tongue movement | 12 | 379 (6 to 24) | 2 (1 to 23) | *p*<0.01 |
| Turkeltaub (2002)[^39^](#_ENREF_39) | Reading | 11 | 160 (6 to 17) | 16 (2 to 33) | *p*< 0.0001 |
| Turkeltaub (2002)[^39^](#_ENREF_39) | Acoustic vs. phonological processing | 23 | 300 (6 to 28) | 4 (1 to 23) | FDR<0.01 |
| Turkeltaub (2002)[^39^](#_ENREF_39) | Categorical phoneme perception | 8 | 123 (6 to 28) | 1 (1 to 23) | FDR<0.01 |
| van der Laan (2011)[^40^](#_ENREF_40) | Food cue reactivity (food vs non food) | 18 | 246 (8 to 25) | 16 (1 to 23) | *p*<0.05 |
| van der Laan (2011)[^40^](#_ENREF_40) | Food cue reactivity (hungry vs satiated) | 5 | 57 (9 to 17) | 2 (3 to 24) | *p*<0.05 |
| van der Laan (2011)[^40^](#_ENREF_40) | Food cue reactivity (high vs low energy food) | 7 | 112 (8 to 25) | 5 (5 to 42) | *p*<0.05 |
| Veldhuizen (2011)[^41^](#_ENREF_41) | Food cue reactivity (taste) | 15 | 169 (7 to 18) | 9 (5 to 40) | *p*<0.05 |
| Wiener (2010)[^42^](#_ENREF_42) | Implicit timing | 12 | 140 (7 to 25) | 1 (1 to 11) | FDR<0.01 |

**Legend:** All meta-analyses included by first author, year of publication, condition, the number of individual fMRI studies included in the meta-analysis, the range of sample sizes for the individual studies included, the range of foci, and designated meta-analytic significance threshold.

*Note***.** Healthy controls except when specified otherwise (e.g., autism spectrum disorder or schizophrenia patients). General age range unless specified otherwise (e.g., children or adolescents). Stimulus condition noted only (except when multiple conditions compared). FDR = false discovery rate. *Uncorrected *P* values given for each foci. No *P* value was greater than 0.05. **Some foci were analyzed at a more conservative *p*-value of 0.005. ***Cerebellum only.

**REFERENCES**

1. Albrecht, J.*, et al.* The neuronal correlates of intranasal trigeminal function-an ALE meta-analysis of human functional brain imaging data. *Brain Research Reviews* **62**, 183-196 (2010).

2. Amanzio, M., Benedetti, F., Porro, C.A., Palermo, S. & Cauda, F. Activation likelihood estimation meta-analysis of brain correlates of placebo analgesia in human experimental pain. *Human Brain Mapping*  (2011).

3. Arsalidou, M., Morris, D. & Taylor, M.J. Converging evidence for the advantage of dynamic facial expressions. *Brain Topography* **24**, 149-163 (2011).

4. Brooks, S.J.*, et al.* Exposure to subliminal arousing stimuli induces robust activation in the amygdala, hippocampus, anterior cingulate, insular cortex and primary visual cortex: a systematic meta-analysis of fMRI studies. *NeuroImage* **59**, 2962-2973 (2012).

5. Brown, S., Gao, X., Tisdelle, L., Eickhoff, S.B. & Liotti, M. Naturalizing aesthetics: brain areas for aesthetic appraisal across sensory modalities. *NeuroImage* **58**, 250-258 (2011).

6. Bzdok, D.*, et al.* ALE meta-analysis on facial judgments of trustworthiness and attractiveness. *Brain Structure & Function* **215**, 209-223 (2011).

7. Caspers, S., Zilles, K., Laird, A.R. & Eickhoff, S.B. ALE meta-analysis of action observation and imitation in the human brain. *NeuroImage* **50**, 1148-1167 (2010).

8. Davis, M.H. & Gaskell, M.G. A complementary systems account of word learning: neural and behavioural evidence. *Philosophical Transactions of the Royal Society of London. Series B, Biological Sciences* **364**, 3773-3800 (2009).

9. Derrfuss, J., Brass, M., Neumann, J. & von Cramon, D.Y. Involvement of the inferior frontal junction in cognitive control: meta-analyses of switching and Stroop studies. *Human brain mapping* **25**, 22-34 (2005).

10. Di Martino, A.*, et al.* Functional brain correlates of social and nonsocial processes in autism spectrum disorders: an activation likelihood estimation meta-analysis. *Biological Psychiatry* **65**, 63-74 (2009).

11. Diekhof, E.K., Geier, K., Falkai, P. & Gruber, O. Fear is only as deep as the mind allows: a coordinate-based meta-analysis of neuroimaging studies on the regulation of negative affect. *NeuroImage* **58**, 275-285 (2011).

12. Eickhoff, S.B.*, et al.* Coordinate-based activation likelihood estimation meta-analysis of neuroimaging data: a random-effects approach based on empirical estimates of spatial uncertainty. *Human Brain Mapping* **30**, 2907-2926 (2009).

13. Gianaros, P.J. & Sheu, L.K. A review of neuroimaging studies of stressor-evoked blood pressure reactivity: emerging evidence for a brain-body pathway to coronary heart disease risk. *NeuroImage* **47**, 922-936 (2009).

14. Houdé, O., Rossi, S., Lubin, A. & Joliot, M. Mapping numerical processing, reading, and executive functions in the developing brain: an fMRI meta-analysis of 52 studies including 842 children. *Developmental science* **13**, 876-885 (2010).

15. Jardri, R., Pouchet, A., Pins, D. & Thomas, P. in Schizophrenia : A Coordinate-Based Meta-Analysis. *American Journal of Psychiatry* **168**, 73-81 (2011).

16. Jirak, D., Menz, M.M., Buccino, G., Borghi, A.M. & Binkofski, F. Grasping language--a short story on embodiment. *Consciousness and Cognition* **19**, 711-720 (2010).

17. Kim, H. Neural activity that predicts subsequent memory and forgetting: a meta-analysis of 74 fMRI studies. *NeuroImage* **54**, 2446-2461 (2011).

18. Krain, A.L., Wilson, A.M., Arbuckle, R., Castellanos, F.X. & Milham, M.P. Distinct neural mechanisms of risk and ambiguity: A meta-analysis of decision-making. *NeuroImage* **32**, 477-484 (2006).

19. Kühn, S. & Gallinat, J. A quantitative meta-analysis on cue-induced male sexual arousal. *The Journal of Sexual Medicine* **8**, 2269-2275 (2011).

20. Kühn, S. & Gallinat, J. Resting-state brain activity in schizophrenia and major depression: A quantitative meta-analysis. *Schizophrenia Bulletin*, 1-8 (2011).

21. Kühn, S. & Gallinat, J. Common biology of craving across legal and illegal drugs - a quantitative meta-analysis of cue-reactivity brain response. *The European Journal of Neuroscience* **33**, 1318-1326 (2011).

22. Laird, A.R.*, et al.* Comparison of the disparity between Talairach and MNI coordinates in functional neuroimaging data: validation of the Lancaster transform. *NeuroImage* **51**, 677-683 (2010).

23. Maisog, J.M., Einbinder, E.R., Flowers, D.L., Turkeltaub, P.E. & Eden, G.F. A meta-analysis of functional neuroimaging studies of dyslexia. *Annals of the New York Academy of Sciences* **1145**, 237-259 (2008).

24. Mar, R.A. The neural bases of social cognition and story comprehension. *Annual Review of Psychology* **62**, 103-134 (2011).

25. Mechias, M.-L., Etkin, A. & Kalisch, R. A meta-analysis of instructed fear studies: implications for conscious appraisal of threat. *NeuroImage* **49**, 1760-1768 (2010).

26. Mohr, P.N.C., Biele, G. & Heekeren, H.R. Neural processing of risk. *The Journal of Neuroscience* **30**, 6613-6619 (2010).

27. Moulton, E.a., Schmahmann, J.D., Becerra, L. & Borsook, D. The cerebellum and pain: passive integrator or active participator? *Brain research reviews* **65**, 14-27 (2010).

28. Petacchi, A., Laird, A.R., Fox, P.T. & Bower, J.M. Cerebellum and auditory function: an ALE meta-analysis of functional neuroimaging studies. *Human Brain Mapping* **25**, 118-128 (2005).

29. Richlan, F., Kronbichler, M. & Wimmer, H. Functional abnormalities in the dyslexic brain: a quantitative meta-analysis of neuroimaging studies. *Human Brain Mapping* **30**, 3299-3308 (2009).

30. Richlan, F., Kronbichler, M. & Wimmer, H. Meta-analyzing brain dysfunctions in dyslexic children and adults. *NeuroImage* **56**, 1735-1742 (2011).

31. Rotge, J.-y.*, et al.* Provocation of obsessive – compulsive symptoms : a quantitative voxel-based meta-analysis of functional neuroimaging studies. *Journal of Psychiatry Neuroscience* **33**, 405-412 (2008).

32. Simmonds, D.J., Pekar, J.J. & Mostofsky, S.H. Meta-analysis of Go/No-go tasks demonstrating that fMRI activation associated with response inhibition is task-dependent. *Neuropsychologia* **46**, 224-232 (2008).

33. Sörös, P., Inamoto, Y. & Martin, R.E. Functional brain imaging of swallowing: an activation likelihood estimation meta-analysis. *Human Brain Mapping* **30**, 2426-2439 (2009).

34. Spaniol, J.*, et al.* Event-related fMRI studies of episodic encoding and retrieval: meta-analyses using activation likelihood estimation. *Neuropsychologia* **47**, 1765-1779 (2009).

35. Spreng, R.N., Mar, R.a. & Kim, A.S.N. The common neural basis of autobiographical memory, prospection, navigation, theory of mind, and the default mode: a quantitative meta-analysis. *Journal of Cognitive Neuroscience* **21**, 489-510 (2009).

36. Spreng, R.N., Wojtowicz, M. & Grady, C.L. Reliable differences in brain activity between young and old adults: a quantitative meta-analysis across multiple cognitive domains. *Neuroscience and Biobehavioral Reviews* **34**, 1178-1194 (2010).

37. Stoodley, C.J. & Schmahmann, J.D. Functional topography in the human cerebellum: a meta-analysis of neuroimaging studies. *NeuroImage* **44**, 489-501 (2009).

38. Takai, O., Brown, S. & Liotti, M. Representation of the speech effectors in the human motor cortex: somatotopy or overlap? *Brain & Language* **113**, 39-44 (2010).

39. Turkeltaub, P.E., Eden, G.F., Jones, K.M. & Zeffiro, T.a. Meta-Analysis of the functional neuroanatomy of single-word reading: method and validation. *NeuroImage* **16**, 765-780 (2002).

40. van der Laan, L.N., de Ridder, D.T.D., Viergever, M.a. & Smeets, P.a.M. The first taste is always with the eyes: a meta-analysis on the neural correlates of processing visual food cues. *NeuroImage* **55**, 296-303 (2011).

41. Veldhuizen, M.G.*, et al.* Identification of human gustatory cortex by activation likelihood estimation. *Human Brain Mapping* **32**, 2256-2266 (2011).

42. Wiener, M., Turkeltaub, P.E. & Coslett, H.B. Implicit timing activates the left inferior parietal cortex. *Neuropsychologia* **48**, 3967-3971 (2010).
